# The expression pattern of *OsDim1* in rice and its proposed function

**DOI:** 10.1038/s41598-019-54898-1

**Published:** 2019-12-06

**Authors:** Henry Akrofi Doku, Shu-Xian Gan, Qian Zhu, Sadia Nadir, Wei Li, Meng-Ting Li, Li Zhou, Cheng-Yun Li, Sang-Gu Kang, Eui-Ho Park, Li-Juan Chen, Dong-Sun Lee

**Affiliations:** 1 Rice Research Institute, Yunnan Agricultural University, Kunming, 650201 Yunnan Province China; 2 Institute of Agricultural Sciences, Xishuanbanna Prefecture, Yunnan Province China; 3 State Key Laboratory for Conservation and Utilization of Bio-Resources in Yunnan, Yunnan Agricultural University, Kunming, 650201 Yunnan China; 4 Department of Biotechnology, Institute of Biotechnology, College of Life and Applied Sciences, Yeungnam University, Gyeongsan, Gyeongbuk 38541 Republic of Korea; 5 Biotechnology Lab Complex, CSIR-Crops Research Institute, Fumesua, Kumasi Ghana; 6 Department of Chemistry, University of Science and Technology Bannu, KPK, Bannu, Pakistan

**Keywords:** Gene expression, Gene expression, Mutation, Mutation, Plant breeding

## Abstract

Development of plant tissues is dependent on numerous factors, including hormone activity, signaling, cell division, and elongation. In plants, *Defective Entry into Mitosis 1* (*Dim1*) homologs are recognized as pivotal in leaf senescence and progress of normal growth, but their role in rice has not been functionally characterized. The findings presented in this paper suggest that *OsDim1* is important in early seedling development, pollen tube elongation, and impacts rice yield components. The gene is expressed in the scutellum, endosperm, embryonic root, shoot, pollen grains and tubes, as well as in several organs of the rice flower. According to the present study findings, RNAi mediated knockdown of *OsDim1* resulted in phytohormonal imbalance, reduced amylase activity, affected differentiation of embryonic root elongation zone tissues, suppressed embryonic root and shoot growth, and impaired pollen tube elongation. In contrast, overexpression of *OsDim1* showed significant growth in embryonic roots and shoots, while it increased culm length, total number of tillers per plant, seed setting rate, and total number of grains per panicle compared to its wild type line. In summary, we propose *OsDim1* plays an important role in seedling growth and pollen tube elongation, and has pleiotropic effects on reproductive tissues.

## Introduction

The *Defective entry into mitosis 1* (*Dim1*) gene family is part of the thioredoxin (TRX)-super family and has been demonstrated to play an essential role in cell cycle progression, particularly during entry into mitosis, and mitotic events, such as segregation of chromosome^1,2^. Nonetheless, unlike typical TRX enzymes, the Dim1 protein family lacks the Cys-X-X-Cys motif^3^ which is primarily used for redox reactions after reduction by NADP-TRX reductase (NTR) and NADPH^4–6^. Thus, the Dim1 proteins possess the common TRX*-*like fold, but they contain Asp-X-X-Cys motifs instead^3,7,8^. However, while *Dim1* is involved in the formation of disulfide bonds, it does not exhibit a typical TRX oxidoreductase activity^3,9^, making its role uncertain. For instance, in human *Dim1* (U5–15KB), the second cysteine (Cys38) residue, along with another cysteine (Cys79), has been suggested to be involved in the formation of disulfide bonds. Yet, a purified recombinant Dim1 in a dithiol-disulfide redox test failed to support this claim^3^. Failure of Dim1 to play the role of typical TRXs despite its involvement in disulfide bonds has also been attributed to the lack of Cys79 conservation, the diminished cell environment, and the reduced stability of the protein in the presence of disulfide^9,10^. Dim1 proteins are critical components of the U5 small nuclear ribonucleoproteins (U5 snRNP). Structurally, Dim1 is considered an essential protein that connects different components of the U5 snRNP^11^. TRX-like Dim1 (U5–15K) interacts with the N-terminus of Prp6 (an snRNP U5 protein), an important element of the spliceosome, to form a U4/U6.U5 tri-snRNP bridge^11,12^.

The biological function of *Dim1* is well preserved in different eukaryotic organisms, including *Schizosaccharomyces pombe*, *Saccharomyces cerevisiae*, slime molds, alveolate plants, *Caenorhabditis elegans*, and mammals. The sequence similarity of the gene across species is about 79% of the total length comprising of 142 amino acids^3,8^. The *yellow leaf specific gene 8* (*YLS8*) was the first gene identified as a *Dim1* homologue in plants and shares 85% and 76% protein sequence similarity with *Homo sapiens* and *S. pombe Dim1*, respectively^1,13^. *YLS8* is involved in leaf senescence, though its exact role remains to be elucidated^13^. Recently, *Dim1* has been proposed to be necessary for the normal growth of soybean^14^. Overexpression of the soybean *Dim1* gene (*GmDim1*) in *Arabidopsis thaliana* promoted early flowering, the growth of multiple shoots and stem elongation^14^. However, the functional role of *Dim1* in rice (*OsDim1*) has not been characterized. The work presented in this article proposes that *OsDim1* supports growth of seedling root and shoot, as well as pollen tube elongation. Our results also suggest that *OsDim1* may contribute to the balance of hormones in the post-ripening phase of seed development, which is associated with seed emergence. This article further suggests that *OsDim1* plays a pleiotropic role in rice reproductive tissues and impacts the grain yield components. The findings yielded by the present study thus improves our current understanding of the role of *Dim1* genes in these processes.

## Results

### Bioinformatic characterization of *OsDim1*

**Figure 1:**
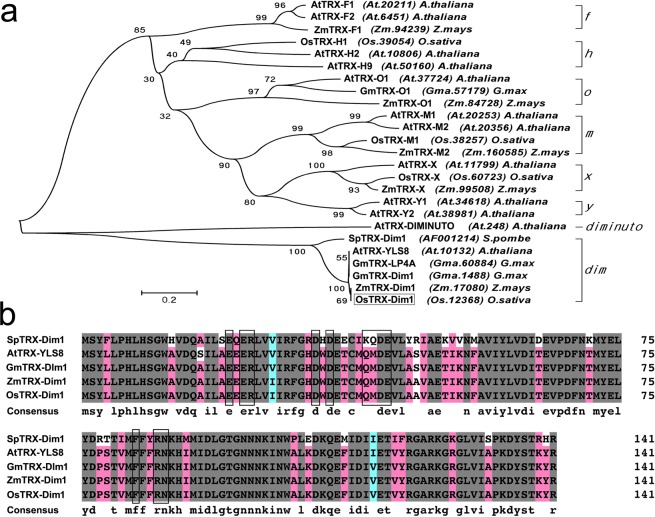
Phylogenetic Tree and Sequence homology of OsDim1 Protein. (**a**) Phylogenetic relationship between OsDim1 protein and some members of the thioredoxin-like super family. OsDim1 protein is indicated in the phylogenetic tree as OsTRX-Dim1 (enclosed in a box) and forms a branch with thioredoxin-like *Zea mays* mitosis Dim1, *Arabidopsis thaliana* YLS8, *Glycine max* Dim1, and *S. pombe* Dim1 proteins at a bootstrap value of 100. The phylogenetic tree was constructed by means of MEGA software (version 5.0) using the neighbor-joining method. Bootstrap value = 0.2 and the text in parentheses denote the accession numbers or Unigene entry codes (https://www.ncbi.nlm.nih.gov/unigene) of the proteins. (**b**) Amino acid sequence alignment of the thioredoxin-like Dim1 proteins that formed a branch with OsDim1 protein at a bootstrap value of 100%, as shown in the phylogenetic tree. The boxes indicate the critical sites of the conserved domains of Dim1 the protein family. The alignment was performed with DNAMAN8.

*OsDim1* (Os.12368, AK063786) is a 794 bp member of the TRX superfamily, located on chromosome 10. It has two exons and encodes a 142-residue protein with a *Dim1* and *TRX* A domain*. Dim1* and *TRX* A are structurally similar due to the thioredoxin-like fold they both possess (https://www.ncbi.nlm.nih.gov/Structure/cdd/wrpsb.cgi?SEQUENCE = 18087886&FULL). The OsDim1 protein shares high sequence similarity, or forms a branch at a bootstrap value of 100%, with Dim1 proteins, including *Zea mays* mitosis Dim1, *Arabidopsis thaliana* YLS8, *Glycine max* Dim1, and *S. pombe* Dim1 (Fig. 1a,b). OsDim1 shares no similarity sequence with thioredoxin isoforms (*f, h, o, m, x, y*) proteins as well as Diminuto protein in Arabidopsis (Fig. 1a). Analysis of the *OsDim1* promoter region (2727 bp) via Plant CARE database indicates presence of 34 *cis*-acting regulatory elements related to various physiological and metabolic processes. These include endosperm expression (Skn-1), circadian control, meristem-specific activation, TATA box, CAAT box, and phytohormone responsive elements, including auxin, abscisic acid, and methyl jasmonate (Supplementary Table S1). *In silico* protein-protein interaction predictions by STRING indicated that OsDim1 proteins interact with putative pre-mRNA processing factor 6, pre-mRNA processing factor 31, WW, and LSM-domain containing proteins. The pre-mRNA processing factors are essential for pre-mRNA splicing, mRNA stability, miRNA biogenesis, and nuclear export^15,16^. WW proteins function as transcription co-activators, whereas LSM-domain containing proteins play a role in mRNA decay pathway^17–20^.

### Expression pattern and sub-cellular localization of *OsDim1*

**Figure 2:**
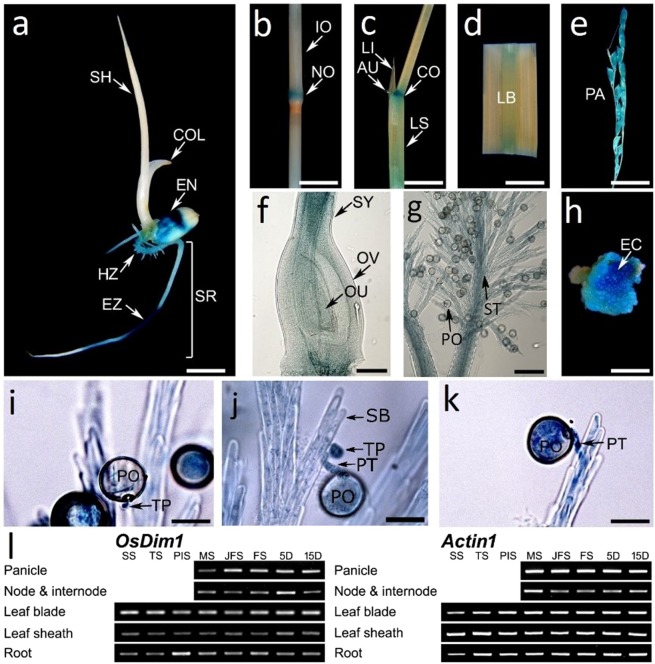
Expression Pattern of *OsDim1* in Different Tissues of *OsDim1*_*pro*_*:GUS* (GUS) Transgenic Rice Plant. (**a**) Histochemical staining assay showing the expression of *OsDim1* in the endosperm (EN) as well as zones of cell elongation (EZ) and root hair (HZ) of the seminal root (SR) of a 5-day-old GUS transgenic rice seedling. Histochemical assay showing expression of *OsDim1* in the nodal septal (NO) (**b**), auricles (AU), collar (CO), leaf sheath (LS) (**c**), leaf blade (LB) (**d**), immature panicle (PA) (**e**), style (SY) (**f**), pollen grains (PO) and stigma (ST) (**g**) at the early reproductive stage of the rice plant. (**h**) Expression of *OsDim1* in the embryogenic callus of GUS transgenic rice. Pollen mean time transit test showing expression of *OsDim1* in the stigma (SB), as well as the pollen tips or the entire pollen tube (PT) during its emergence and elongation at three minutes after pollination (3MAP) (**i**), 5MAP (**j**) and 30 MAP (**k**). (**l**) RT-PCR analysis of *OsDim1* in comparison with the rice *β-actin* gene. *OsDim1* expression was detected in the panicles, nodes and internodes, leaf blade, leaf shoot and root of the rice plant at the seedling stage (SS), tiller stage (TS), panicle initiation stage (PIS), meiotic division phase of the rice panicle (flower) development (MS), just before the flowering stage (JFS), flowering stage (FS), five days after pollination (5 D), and fifteen days after pollination (15 D) of the rice plant. Abbreviations: COL, coleoptile; LI, ligule; OV, ovary; OU, ovules, SH, shoot. TP, tip of pollen tube. Full length gels of the RT-PCR results are displayed in Supplementary Fig. S1.

To gain insight into the function of *OsDim1* in rice, its tissue-specific expression was studied using RT-PCR. The panicle, node and internode, leaf blade, leaf sheath, and roots were sampled during the vegetative, reproductive, and early ripening stages of rice development. *OsDim1* expression was detected in all tissues and across developmental stages (Fig. 2l). Next, the *OsDim1* promoter was fused to the GUS reporter to produce an *OsDim1*_*pro*_*:GUS* construct, which was subsequently used to transform the LiyuB wild type (WT) rice plant.

**Figure 3:**
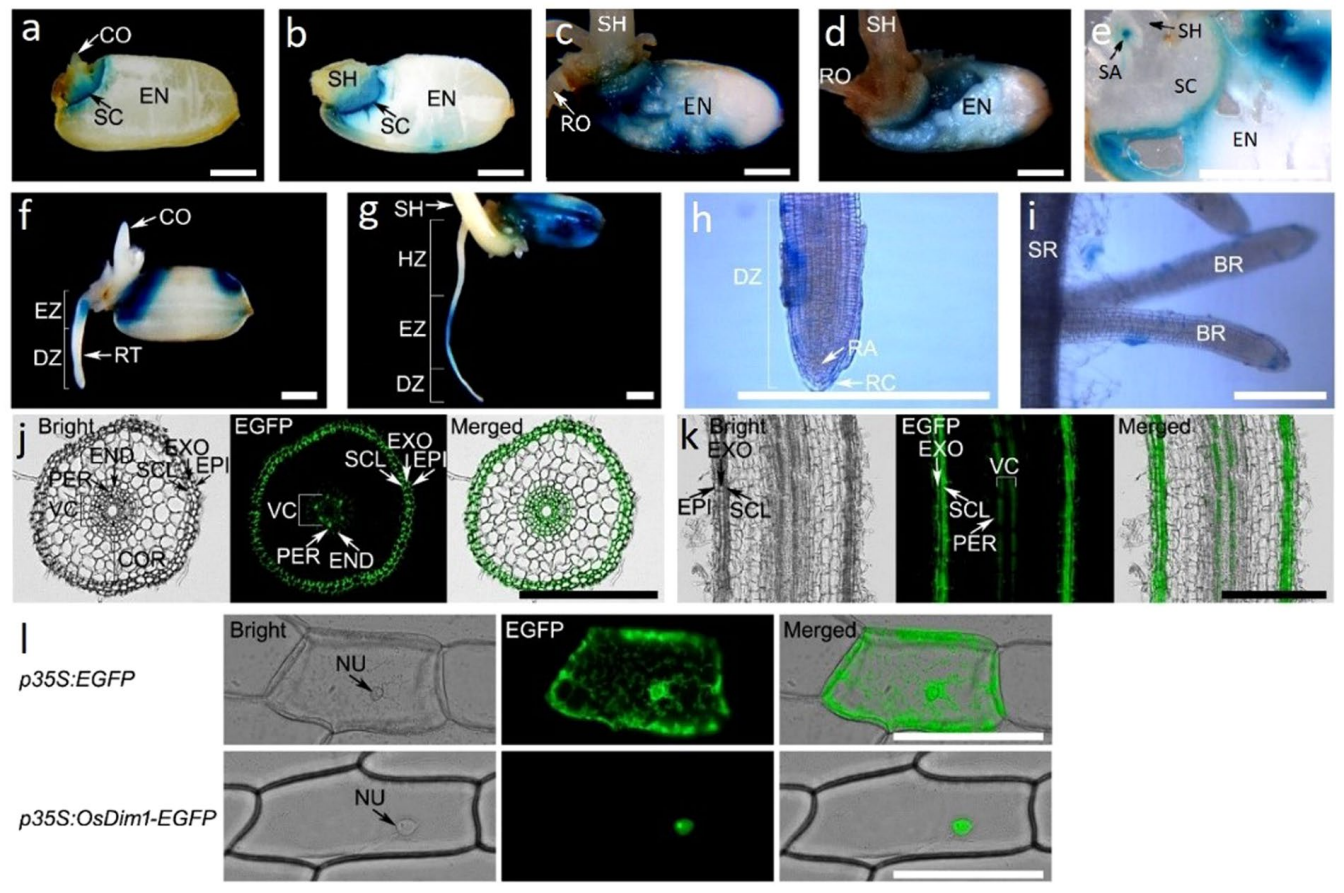
Expression Pattern of *OsDim1* by *β*-Glucuronidase and Enhanced Green Fluorescent Protein (EGFP). Histochemical staining showing expression of *OsDim1* in the internal structure of the endosperm of two (**a**), three (**b**), five (**c**) and seven (**d**) days old *OsDim1*_*pro*_*:GUS* (GUS) transgenic rice seedlings respectively. (**e**) Histochemical staining showing *OsDim1* expression in the shoot apex of a 5-day-old GUS transgenic rice seedling. Histochemical staining showing the expression of *OsDim1* on the external structure/layer of the endosperm of 2 (**f**) and 7 (**g**) days old GUS transgenic rice seedlings, respectively. Histochemical staining showing *OsDim1* expression in the seminal root DZ (**h**) and branch root (**i**) of 5-day-old GUS transgenic rice seedlings, respectively. (**j**) EZ cross-section of the seminal root of a 5-day-old GUS transgenic rice seedling showing *OsDim1* fluorescence signals in the exodermis (EXO), sclerencymatous cells (SCL), endodermis (END), pericycle (PER) and vascular cylinder (VC). (**k**) Vertical section of seminal root EZ of a 5-day-old GUS transgenic rice seedling showing *OsDim1* fluorescence signals. (**l**) Fluorescence microscopy showing subcellular localization of *OsDim1* in onion epidermal cells. Top panel: Control EGFP (*35S*_*pro*_*:EGFP*) fluorescence signals were identified in the nucleus (NU) and cytoplasm of onion epidermal cells by bright fields, EGFP, and merged images, respectively. Bottom panel: *OsDim1* fused to EGFP (*35S*_*pro*_*:OsDim1-EGFP*) signals were observed only in the nucleus of onion cells by bright fields, EGFP, and merged images, respectively. Bar length = 200 µm. Abbreviation: COR − cortex.

The *OsDim1*_*pro*_*:GUS* transgenic rice plants generated exhibited similar phenotypic characteristics to those of their WT. Histochemical staining showed expression of the *OsDim1* in the *OsDim1*_*pro*_*:GUS* transgenic rice seedlings, specifically in the endosperm, and the various zones of the seminal root—cell elongation (EZ) and cell maturation/root hair (HZ)— between day 2 and day 7 after seeding (Fig. 2a). At the mitotic division phase of the rice panicle development (early reproductive stage), *OsDim1* expression was observed in various *OsDim1*_*pro*_*:GUS* transgenic rice plant organs, including the nodal septal, auricle, leaf collar, leaf sheath, leaf blade, and immature panicle (Fig. 2b–e). Similar *OsDim1* expression patterns were also detected in the nodal septal, leaf sheath, auricle, leaf collar, leaf blade, and the fully developed panicles in the flowering phase (early ripening stage) of the rice plant development. Expression of *OsDim1* was observed in the style, stigma, and pollen grains of the *OsDim1*_*pro*_*:GUS* rice flower (Fig. 2f,g), as well as in the embryonic callus (Fig. 2h). Pollen mean time transit test showed expression of *OsDim1* in the tip of the pollen tube or the entire pollen tube during its emergence and elongation at 3, 5 and 30 mins after pollination (MAP), denoted respectively as 3 MAP, 5 MAP, and 30 MAP (Fig. 2i–k). In the rice seedling, *OsDim1* expression was observed in the endosperm of the growing seedlings to increase or spread as germination proceeded. Thus, *OsDim1* expression was first observed in the region of the rice scutellum connecting the embryo and the endosperm two days after seeding (2 DAS). Subsequently, *OsDim1* expression progressively spread from the scutellum region to about 1/2 and at least 3/4 of the endosperm at 5 DAS and 7 DAS, respectively. Dissection of the endosperm of the seedlings harvested during the 2–7 DAS period indicated that *OsDim1* expression was occurring deep in the endosperm (Fig. 3a–d), rather than solely near the pericarp (Fig. 3f,g). During the dissection of the rice seed, the expression of *OsDim1* in the shoot apex was also noted (Fig. 3e).

Owing to the expression of *OsDim1* in the seminal root of the rice seedling, the gene expression in the zone of cell division (DZ) of the seminal root, as well as the branch root (BR) or root hair of a 5-day-old rice seedling was examined through paraffin sectioning. The results obtained revealed ubiquitous expression (in blue spots) of *OsDim1* throughout the tissues of the DZ—including the meristematic or apical initials of the root apex (RA)—and BR (Fig. 3h,i). To further explore *OsDim1* expression in the rice root, *OsDim1* enhanced green fluorescent protein (*OsDim1*_*pro*_*:OsDim1-EGFP*) rice transgenic plants were created. Internal structure (cross and vertical sections) of the seminal root’s EZ of 5-day-old *OsDim1*_*pro*_*:OsDim1-EGFP* rice seedlings showed expression (fluorescent signals) of the gene in the exodermis (EXO), sclerencymatous cells (SCL), endodermis (END), pericycle (PER), and the entire region of the vascular cylinder (VC), as shown in Fig. 3j,k. *OsDim1* fluorescent signal was not detected in the cortex (COR). The aforementioned findings suggest that *OsDim1* may play a role in the embryonic root development during germination of rice seedlings.

To examine the sub-cellular localization of *OsDim1*, an *OsDim1*-EGFP construct with a *Cauliflower mosaic virus* (*CaMV*), 35 S promoter (*35S*_*pro*_*:OsDim1-EGFP*) was created. This construct was transiently transfected into onion epidermal cells. Analysis of transformed onion epidermal cells revealed *OsDim1-*EGFP signals in the nucleus (Fig. 3l).

### Suppression of *OsDim1* impairs growth of embryonic root and shoot

**Figure 4:**
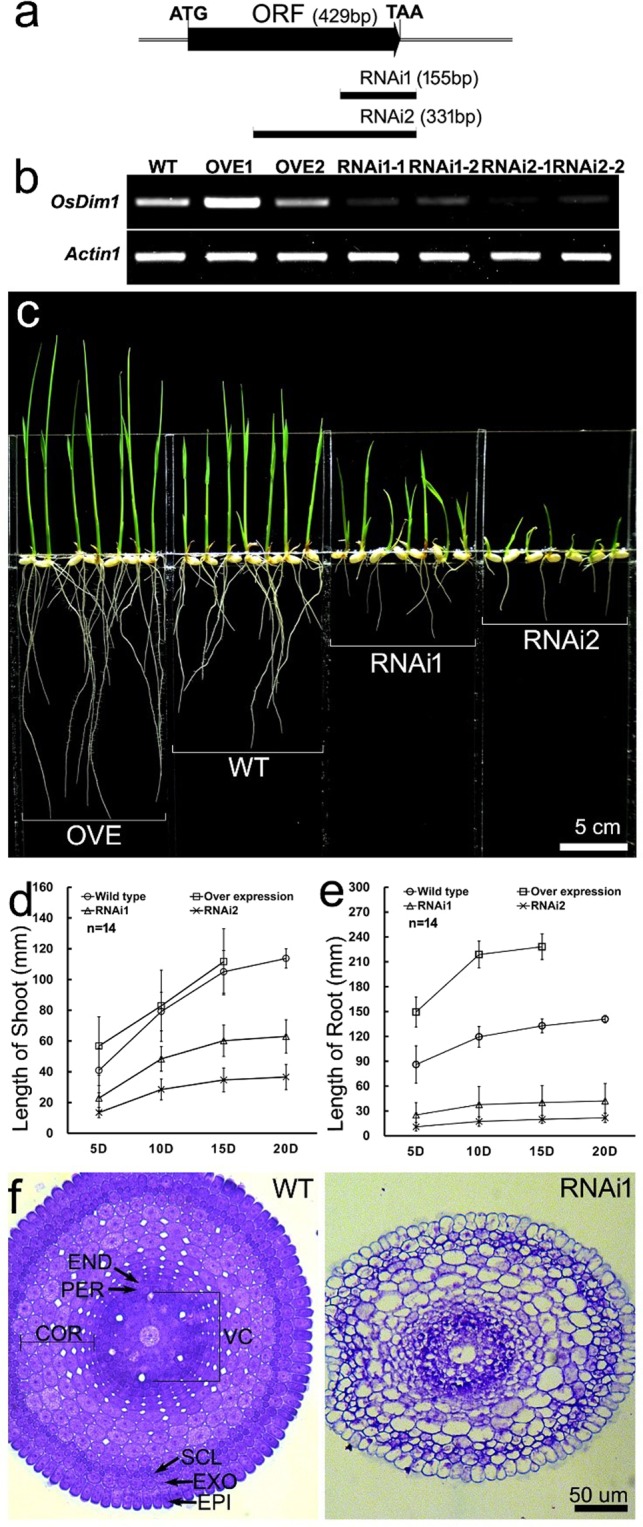
Comparison of the Morphology and Internal Structure of WT and *OsDim1* Transgenic Rice Seedlings. (**a**) Molecular cloning of RNAi constructs. The diagram depicts the *OsDim1* ORF length (429 bp), and the fragment length or cloning position for RNAi 1 (155 bp) and RNAi 2 (331 bp) transgenic rice development. (**b**) RT-PCR analysis showing low levels of expression of RNAi mediated knock down lines compared to the OVE and WT lines, with the rice *β-actin* gene as an internal standard to normalize the expressions. Full length gels of the RT-PCR results are displayed in Supplementary Fig. S2. (**c**) Phenotypic difference of the root and shoot length between the WT and *OsDim1* transgenic rice lines after ten (10) days of seeding. Estimation of the differences in shoot (**d**) and root (**e**) length between the OVE, WT and RNAi rice lines. Shoot and root length in the WT and RNAi lines were assessed over a period of 20 days, while the period was shortened to only 15 days for the OVE line. (**f**) Comparison of the internal structure (cross-section) of the seminal roots of 5-day-old WT and RNAi rice seedlings. Abbreviations: OVE, *OsDim1* overexpression transgenic seedlings; RNAi1, OsDim1-RNA interference (RNAi) transgenic line 1 rice seedlings; RNAi2, RNAi transgenic line 2 rice seedlings; WT, LiyuB wild type seedlings.

Two RNAi binary vectors (*35S*_*pro*_*:ODim1-RNAi1* and *35S*_*pro*_*:OsDim1-RNAi2*) were constructed (Fig. 4a) to further characterize *OsDim1* in rice. Several independent transformants expressing similar characteristics were generated using the *35S*_*pro*_*:OsDim1-RNAi1* and *35S*_*pro*_*:OsDim1-RNAi2* binary vectors, and subsequently screened. At the T_2_ stage, we selected one independent transformant, with moderate expression, each from *35S*_*pro*_*:OsDim1-RNAi1* and *35S*_*pro*_*:OsDim1-RNAi2* transgenic lines and designated them as RNAi 1 and RNAi 2 respectively in the present study. Thus, *OsDim1*-RNAi (RNAi) transgenic plants with low expression levels of the gene were selected for evaluation (Fig. 4b). RNAi T_2_ lines were grown for 20 days alongside the wild type (as a control) for comparison. Shoot and root lengths of the germinating rice seedlings were recorded every five days. The RNAi transgenic lines showed significant reduction in seedling shoot and root length compared to the WT across all time points (Fig. 4c–e). Analysis of internal structures of the seminal root EZ of 5-day-old seedlings revealed structural differences between the WT and RNAi tissues (Fig. 4f). The internal structure of the EZ of 5-day-old WT seedlings’ seminal roots comprised of well-developed EXO, SCL, END, PER, and VC tissues, with their boundary layers well differentiated and defined. Additionally, *OsDim1* was ubiquitously expressed (blue dots) in almost all tissues of the WT seminal root EZ. On the other hand, the EZ of 5-day-old RNAi seedlings’ seminal root showed poor differentiation of EXO, SCL, END, PER, and VS tissues. Moreover, the boundary layers of the individual tissues were not properly defined and lacked expression of *OsDim1* in some of the tissues.

### *OsDim1* overexpression promotes root and shoot growth during rice germination

Transgenic plants characterized by *35S*_*pro*_*:OsDim1* overexpression were also generated through the introgression of a *35S*_*pro*_*:OsDim1* binary vector into the WT. The *OsDim1*-OVE (OVE) T_2_ plants derived from one OVE T_0_ line were grown alongside the WT, which served as controls. Shoot and root length were recorded over 15 days at 5-day intervals. In the OVE rice seedlings, a significant root length increase was observed compared to the WT (Fig. 4c–e). Specifically, the average root length in the OVE transgenic seedlings was 149.4 mm, 218.8 mm, and 228.2 mm on Day 5, 10, and 15, respectively, whereas 86.0 mm, 119.4 mm, and 132.7 mm were obtained for the WT (Fig. 4d,e). OVE shoot length was also significantly longer on Day 5 and 15 than that measured for the WT.

**Figure 5:**
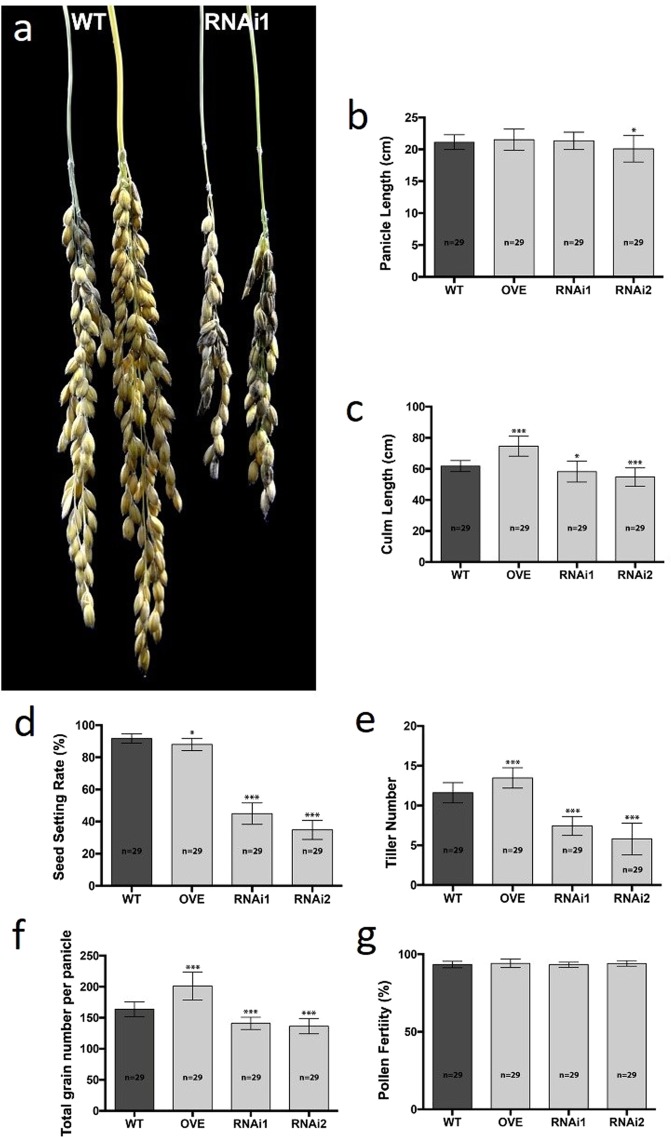
Assessment of Morphological Traits of the WT, OVE, and RNAi Lines. Phenotypic discrepancies in panicle length (**a**,**b**), culm length (**c**), seed setting rate (**d**), tiller number (**e**) and total number of grains per panicle (**f**) between the WT, OVE and RNAi rice lines. No significant differences were observed in the pollen fertility (**g**) between the WT, OVE and RNAi rice lines. Asterisks indicate statistically significant differences (*P < 0.05) and statistically highly significant differences (**P < 0.01 and ***P < 0.001). Error bars indicate standard deviation (±SD). Statistical analysis was performed by one way- ANOVA using the program PRISM 6.0.

The OVE, WT and RNAi seedlings that survived after germination were transplanted and monitored throughout their growth. No significant differences were observed in the panicle length between the WT and the OVE. Differences were observed in the panicle length between WT, and RNAi (PL; Fig. 5a,b), but these were not statistically significant in all cases, particularly the WT and RNAi1 lines. Notably, the OVE plants at differential growth stages of their development revealed significant differences or increases in phenotypic traits, including culm length (CL; Fig. 5c), seed setting rate (SSR; Fig. 5d), total number of tillers per plant (TNT; Fig. 5e), and total grain per panicle (TGP; Fig. 5f) in comparison with the WT. In contrast, significant reduction in phenotypic traits such as CL, SSR, TNT and TGP (Fig. 5c–f), were revealed in the RNAi transgenic plants in comparison to the WT.

### Repression of *OsDim1* impedes elongation of pollen tube

**Figure 6:**
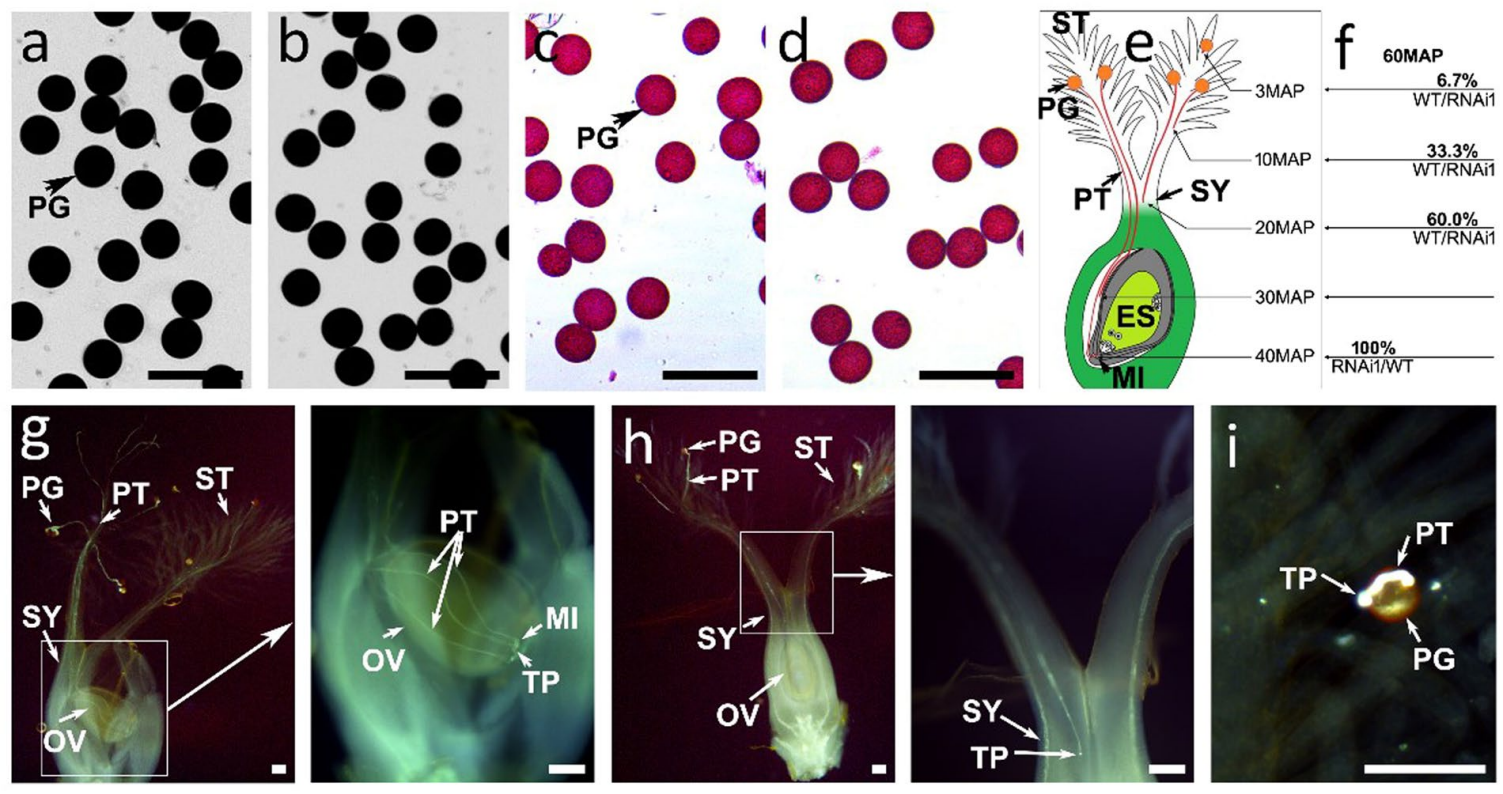
Evaluation of Pollen Fertility, Viability, Germination, and Elongation of Pollen Tube of WT and RNAi Transgenic Rice Lines. Comparison of pollen fertility between WT (**a**) and RNAi (**b**) by I_2_-KI assay. Comparison of pollen viability between WT (**c**) and RNAi (**d**) by Alexander’s staining method. (**e**) A diagram summarizing the findings of the pollen mean time transit test of WT pollen and the growth of its pollen tube. Self-pollinated WT flower (WT/WT) developed pollen tubes that reached the style in 20 mins and penetrated the embryo sac at 40 MAP. (**f**) A diagram summarizing the effect of reciprocal crosses between WT and RNAi flowers for 60 mins. A cross-pollination between 30 WT pollen and 30 RNAi female flowers (RNAi/WT) resulted in germination of all WT pollen (100%), which subsequently developed pollen tubes that elongated and penetrated the embryo sac at 60 MAP. A reciprocal cross between 30 RNAi pollen and 30 WT female flowers (WT/RNAi) resulted in germination of only 60% of the RNAi pollen, which subsequently produced pollen tubes that elongated slowly and reached the style at 60 MAP. (**g**) A demonstration of WT pollen that grew successfully on the RNAi stigma with the tip of its pollen tube in the embryo sac at 60 MAP. The section of (**g**) enclosed in a square is shown next to the image. (**h**) A demonstration of one of the 60% RNAi pollen tubes that reached the WT style at 60 MAP. The section of (**h**) enclosed in a square is shown next to the image. (**i**) A demonstration of one of the 40% RNAi pollen grains on the WT stigma that produced pollen tubes but did not elongate. Bar length = 100 μm.

Owing to the *OsDim1* expression in the pollen grain and tube, the effect of the gene in these tissues when knocked down was assessed. The findings revealed no significant differences in pollen fertility (PF; Figs. 5g, 6a,b) and viability (Fig. 6c,d) between the WT and the RNAi transgenic plants. To examine the effects of gene knockdown on pollen tube growth, a pollen mean time transit test was conducted to compare the WT and the RNAi pollen efficacy in relation to their germination, elongation, and penetration of the embryo sac. The pollen germination and pollen tube elongation of 30 self-pollinated flowers were examined for both WT and RNAi lines. In all self-pollinated WT rice flowers, all pollen grains (100%) germinated normally on the stigma and developed pollen tubes. The developed pollen tubes reached the style in 20 MAP and penetrated the embryo sac at 40 MAP (Fig. 6e). On the other hand, in all self-pollinated RNAi flowers, pollen germination was normal, but elongation of most pollen tubes was abnormal. A reciprocal crossing between the WT and RNAi flowers was subsequently performed to investigate the reasons behind the abnormal pollen tube elongation observed in the RNAi flowers. A cross-pollination between 30 RNAi female flowers and WT pollen (RNAi1/WT) resulted in 100% germination, as well as normal elongation of pollen tubes, and successful penetration of the embryo sac, albeit at 60 MAP (Fig. 6f,g). In contrast, cross-pollination between 30 WT female flowers and RNAi1 pollen (WT/RNAi1) yielded two notable findings. First, 60% of the RNAi1 pollen germinated normally and subsequently developed pollen tubes, which elongated slowly. For instance, the RNAi pollen tubes developed on WT stigmas took about 60 MAP to merely reach the style and were unable to proceed efficiently to the embryo sac (Fig. 6f,h). The remaining 40% of the RNAi pollen germinated on the WT stigmas and produced pollen tubes that could not elongate (Fig. 6i). These observations indicate that *OsDim1* suppression affects pollen tube elongation and its ability to reach the embryo sac.

### Correlation between *OsDim1*, phytohormones and α-amylase

**Figure 7:**
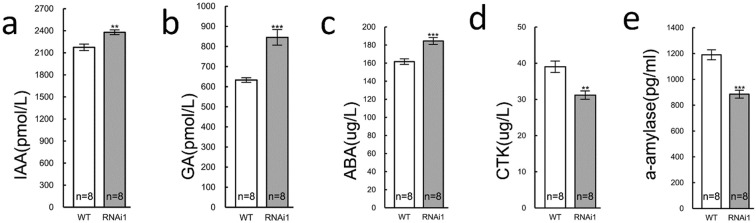
Difference in IAA (**a**), GA (**b**), ABA (**c**), CTK (**d**) and α-Amylase (**e**) concentrations between 5 days old WT and RNAi rice seedlings. Error bars indicate standard deviation (±SD). Asterisks indicate statistically significant differences (*P < 0.05) and highly significant differences (**P < 0.01 and ***P < 0.001). Abbreviations: ABA, abscisic acids; CTK, cytokinin; D, days old; ES, embryo sac; GA, gibberellin; IAA, indole acetic acid, MI, micropyle; OV, ovary, OVE, *OsDim1* overexpressed transgenic line; PG, pollen grain; PT, pollen tube; ST, stigma; SY, style; WT, wild type rice. Statistical analysis was performed by one way- ANOVA using the program PRISM 6.0.

As a part of the present study, whether *OsDim1* is active in phytohormone responsive pathways required for development of the embryonic root and shoot during germination was investigated. For this purpose, indole acetic acid (IAA), gibberellic acid (GA), abscisic acid (ABA), cytokinin (CTK), and α-amylase activities in 5-day-old RNAi and WT seedlings were quantified. The results revealed that the 5-day-old RNAi seedlings had significantly greater levels of IAA (9.4%), GA (33.6%), and ABA (14.1%), which were accompanied by a significant reduction in CTK (20%) and amylase (25.6%) activity compared to the WT plants (Fig. 7a–e). Thus, it can be posited that knockdown of *OsDim1* altered the normal levels of phytohormones and affected the activities of α-amylase required for seed germination and/or seedling development.

## Discussion

In the present study, the sequence of *OsDim1* was analyzed, and its expression pattern was characterized, revealing evidence of subcellular localization, as well as consequences of under- and over-expression. When considered jointly, the results reported in this work demonstrate that *OsDim1* may be important in the consumption or digestion of stored food and more importantly supports embryonic root and shoot growth during the early stages of seed germination. Normally, the onset of germination involves reduction activities by members of the TRX isoform *h* family, as well as activation of hydrolytic enzymes^21–26^. This results in the catabolism of food reserves in the endosperm near the scutellum and mobilization of nutrients to the embryo. Digestion of stored food reserves progresses from the area near the embryo on the ventral side of the seed, extending dorsally downward, and upward to the apex of the endosperm^27^. In the investigated specimens, *OsDim1* expression commenced in the region of the scutellum connecting the embryo and starch reserves of the endosperm. It gradually spread to the apex of the endosperm during the 2−7 day period after seeding (Fig. 3a–d). Overall, the expression pattern of *OsDim1* in the rice seedling embryo and endosperm suggests that this gene generally supports the mobilization and digestion of the stored food during early embryonic tissue development in the seed germination phase.

Members of the *Dim1* gene family generally belong to the TRX superfamily and are involved in the formation of disulfide bonds, but do not participate in redox reaction^3,9,10^. Thus, digestion of nutrients reserved in the endosperm required for seed germination has been solely associated with the TRX *h* family, which has a typical TRX oxidoreductase activity^21,22,26,28,29^. The phylogenetic analysis findings (Fig. 1a) also revealed that OsDim1 shares no protein sequence relationship with TRX h and the other isoforms (*f, h, o, m, x, y* and *z*). These results indicate that *OsDim1* may participate in the formation of the disulfide bonds necessary for seed germination, but is not likely to function in redox activity, even though its expression pattern matches that of the redox-related genes.

Analysis of α-amylase activity in the RNAi seedlings indicated that the level of amylase was significantly lower compared to the WT (Fig. 7e). Since α-amylase is the most active starch digestion enzyme^30–32^, it can be posited that the reduced growth rate of RNAi seedlings over the 5-day period is also related to inadequate starch digestion.

In the present study, *OsDim1* expression was also observed in the various zones of the seminal root (Figs. 2a,3g–k) of developing rice seedlings, particularly the DZ and EZ. Assessment of DZ and BR of the rice seedling seminal roots revealed ubiquitous expression of *OsDim1* (Fig. 3h,i). The DZ contains the root apical meristem, which undergoes constant mitotic cell division and subsequently leads to the formation of epidermis, cortex, and the central stele^27^. The central stele consists of VC and PER^33^. In the EZ of the rice seminal root, *OsDim1* fluorescent signals were also detected in the central stele (VC and PER), END, EXO, and SCL. Examination of the internal structure of RNAi seminal root EZ clearly revealed poor development or abnormal differentiation of VC and PER, END, EXO, and SCL tissues (Fig. 4f). Consequently, in the RNAi phenotypes, embryonic root development was impaired compared to the WT (Fig. 4c–e). In contrast to the RNAi lines, *OsDim1* overexpression resulted in significantly longer seedling roots compared to WT. Thus, these expression patterns of *OsDim1* suggest that the gene may play a role in the normal development of the embryonic root. Additionally, *OsDim1* was expressed in the rice scutellum (Fig. 3a–d,f) which normally undergoes metamorphosis to produce the cotyledon or embryonic leaf (shoot)^32^. *OsDim1* was also expressed in the shoot apex (Fig. 3e) of rice seedlings, which contains leaf apical meristem for shoot development. Knockdown and overexpression of *OsDim1* also caused significant reduction and increase in shoot length, respectively, compared to the WT. When these findings are examined together, the expressions pattern of *OsDim1* in emerging rice seedlings suggest that this gene has a potential role in the breakdown of reserved food nutrients in the seed endosperm and, more importantly, in root and shoot growth during their early seedling development.

The results obtained as a part of this investigation showed that *OsDim1* is important for the elongation of the pollen tubes (Fig. 6e–i). Although the difference in the rate of pollen fertility and viability between the WT and the RNAi lines was not statistically significant, elongation of the RNAi pollen tubes was greatly affected. Two observations pertaining to the elongation of the fertile or viable RNAi pollen are particularly noteworthy. First, 60% of the fertile RNAi pollen in the WT/RNAi crosses developed pollen tubes that elongated slowly and took about 60 MAP to reach the style. In contrast, 60 MAP were needed for the wild type pollen to reach the embryo in the RNAi/WT crosses. Additionally, 20 MAP were needed for the self-pollinated wild type lines to reach the style followed by 40 MAP to penetrate the embryo sac. This difference implies that more time is required for the elongated RNAi pollen tubes to reach the embryo sac, and there is a high possibility that some of the pollen tubes may not be able reach the micropyle or penetrate the embryo sac. Second, the remaining 40% of the RNAi fertile pollen germinated, but produced pollen tubes that could not elongate. This finding suggests that these pollen tubes may not contribute to seed production, since the male nuclei and vegetative nucleus contained in these tubes would not be able to reach the female flower for fertilization. Thus, the poor SSR in the RNAi lines [RNAi1 (45.0 ± 5.5%) and RNAi2 (34.8 ± 5.2%)] may be attributed primarily to the inadequate elongation of the RNAi pollen tubes to reach the female flower for fertilization.

Biologically, the elongation zone in the apical dome of the pollen tip is the main factor that increases the pollen tube length^34^. The pollen tube elongation by its tip is similar to the enlargement of other tissues that grow through their tips, such as root hair and fungal hyphae, whose mechanism of cell enlargement and expansion is different from that observed in other plant cells^34^. Since *OsDim1* expression was found in the both embryonic roots and the tip of pollen tube (Figs. 2i–k, 3f–i), and its RNAi knocked down affected the growth of root as well as the elongation of pollen tube, it is likely that *OsDim1* is vital in these tissues.

The study revealed increased levels of IAA, GA, and ABA (Fig. 7a–c) in 5-day-old seedlings of the RNAi lines compared to the wild type, whereas a reverse trend was observed in CTK concentration (Fig. 7d). The quantified phytohormonal concentrations in the 5 days RNAi seedlings suggests that the aforementioned hormones affected its phenotypic characteristics. First, it is widely established that, during seed germination, embryo development depends largely on upregulation of GA signaling/biogenesis and downregulation of ABA signaling^35–37^. The high ABA concentration in the RNAi lines observed in the present study suggests that ABA biogenesis and/or catabolism was not altered as expected. The increased ABA concentration could potentially be attributed to the high IAA concentration in the RNAi seedlings. Increased IAA content induces the release of auxin receptor factors 10 (ARF10) and 16 (ARF16) to activate the main transcription factor (AB13) for ABA signaling for seed dormancy maintenance, which could contribute to poor germination^38^. Investigation by Belin *et al*.^39^, also revealed that ABA suppresses embryo elongation through IAA signaling. Thus, poor seedling germination could have resulted from an elevated ABA to GA ratio.

Additionally, CTK promotes division of cells in vascular tissues, hypocotyl/shoot growth, and root growth during embryo development^40–42^. Low CTK concentration (Fig. 7d) in the RNAi seedlings observed in the present study suggests that the level of this hormone may be too low to promote embryo shoot/hypocotyl development. Considered together, high GA, IAA, and ABA levels, along with low CTK levels, suggests that disrupted hormone antagonism may partly explain the reduced growth phenotypes of the RNAi seedlings.

Finally, the phenotypic features observed in *OsDim1*-OVE transgenic lines suggest that *OsDim1* may have a potential role in normal plant growth. Physiologically, the superior phenotypic traits observed in OVE seedlings and mature plants relative to its wild counterpart would require active stem cell proliferation in the root apical meristem (RAM) and shoot apical meristem (SAM), which are important determinants in the building of a flowering plant and its patterning. The expression of *OsDim1* in many rice organs is consistent with its homologues in *Arabidopsis* and soybean, which were found by other authors to be expressed in various organs, including root, flower, stem, seeds, nodules and leaves^13,14^. Thus, *OsDim1* gene product may be crucial for rice plant cell life and may also play a constitutive role in plant organs, as demonstrated by its expression in many rice tissues.

### Conclusion

In conclusion, we propose that *OsDim1* is a gene that plays a role in seedling root and shoot growth, as well as elongation of pollen tube. It also exhibits a pleiotropic effect on rice reproductive tissues to impact grain yield.

## Methods

### Gene cloning, vector constructs and development of *OsDim1* transgenic plants

To study the expression pattern of *OsDim1*, a 3.4 kb genomic DNA fragment upstream the promoter region of *OsDim1* was fused to the *GUS* reporter gene and then sub-cloned into DTV1 (modified pCAMBIA1305.2 without enhancer) to produce the *pOsDim1:GUS* construct. To support the assessment of *OsDim1* expression pattern, the enhanced green fluorescent protein (EGFP) reporter was fused to the *OsDim1* promoter and was subcloned into DTV1 (modified pCAMBIA1305.2 without enhancer) to create *OsDim1*_*pro*_*:EGFP* construct. To construct the *OsDim1-*RNAi vector (*35S*_*pro*_*:OsDim1-RNAi)*, an intron fragment containing 85 bp was used as the linker. Using this approach, two RNAi vectors were created, thus RNAi1 and RNAi2 vectors were constructed at the 155 bp and 331 bp cloning positions, respectively, of the *OsDim1* ORF length. To create the overexpression constructs (*35S*_*pro*_*:OsDim1-*OVE), the *OsDim1* cDNA sequence was inserted into EGFP pCAMBIA1302 vectors driven by CaMV35S promoter and *OsDim1* promoter respectively. Each construct was separately mobilized into *Agrobacterium tumefaciens* EHA105 strain, and was incorporated into wild type (WT) rice LiyuB (*O. sativa* ssp. *japonica*) embryonic calli, and various T_0_ transformants [*OsDim1*_*pro*_*:GUS*, *OsDim1*_*pro*_*:OsDim1-EGFP*, *35S*_*pro*_*:OsDim1-RNAi* (RNAi) and *35S*_*pro*_*:OsDim1-OVE* (OVE) transgenic rice plants] were subsequently generated. Transgenic plants were selected by hygromycin resistance and subsequently transplanted. All transgenic materials were examined in the T_0_, T_1_ and T_2_ generations using 10 to 30 independent plants.

### Gene expression analysis by RT-PCR

Extractions and purifications of total RNA from plant tissues at different developmental stages were performed using TRIzol reagent. cDNA was synthesized from 2 mg of total RNA according to the manufacturer’s recommendations. RT-PCR was performed using gene-specific primers (Supplementary Table S2) at 28 reaction cycle with the rice *β-*actin1 (LOC_Os03g50885) primer as an internal standard to normalize the expression of the tested genes.

### *β*-Glucuronidase (GUS) staining assay

GUS assay was conducted as previously demonstrated^43,44^ with slight modification. Tissues at different developmental stages were isolated from *OsDim1*_*pro*_*:GUS* transgenic plants and immediately immersed in 90% acetone at −20 °C for 20 mins. Subsequently, the tissues were rinsed three times with rinsing solution [0.5 mM K_3_Fe (CN)_6_, 0.5 mM K_4_Fe(CN)_6_ 3H_2_O and 50 mM NaPO_4_ buffer (pH 7.2)]. The tissues were further incubated in GUS staining solution [0.1% Triton X-100, 2 mM X-Gluc, 0.5 mM K_3_Fe (CN)_6_, 0.5 mM K_4_Fe (CN)_6_ 3H_2_O and 50 mM NaPO_4_ buffer (pH 7.2)] at 37 °C overnight, fixed in FAA solution, and subsequently bleached with 75% ethanol for observations under a microscope.

### Freeze dry sectioning and analyses of sample tissues

To examine *OsDim1* expression in the internal structure of the rice seedlings, *OsDim1*_*pro*_*:GUS* transgenic plant seeds were grown for two, three, five, and seven days. Next, histochemical or GUS staining was performed on these day-old seedlings, as described above. The embryonic roots (radicle) and portions of the shoots of the 2, 3, 5 and 7-day-old GUS stained seedlings were excised. The seedling tissues were horizontally implanted in embedding medium (Tissue-Tek, Sakura company, #4583) and were subsequently freeze-dried. Semi-thin sections (7 μm) of the embedded organ were cut with a microtome until half of the seedlings were excised. Images of the sectioned seedlings were captured and documented.

### Paraffin sections

To analyze embryonic root elongation zone (EZ), several seminal root samples of 5-day-old seedlings of WT and RNAi plants were separately collected. The EZ of WT and RNAi seminal roots were excised and immediately fixed in cold GA-PFA solution [2.5% glutaraldehyde, 2% paraformaldehyde and 50 mM PIPES (pH 7.2)], before being stored at 4° C overnight, as previously described^45^. Next, the samples were removed, dehydrated in a graded ethanol series (30%, 50%, 70%, 80%, 90%, 95%, and 100% [v/v]) for 10 mins in each gradient at room temperature, and were subsequently embedded in paraffin. Semi-thin sections (7 μm) of the embedded organ were sectioned with a microtome and then stained with Toluidine Blue O. The samples were observed under a Leica microscope.

### Subcellular localization of *OsDim1*

To determine the subcellular localization of *OsDim1*, an *OsDim1*-EGFP fusion construct under the control of the 35 S promoter (*35S*_*pro*_*:OsDim1-EGFP*) was transiently transfected into onion epidermal cells, as previously described^46^. The expression was observed by fluorescence microscopy (Life EVOS digital inverted microscopy FL, EVOS FL, Thermo Fisher Scientific Inc., USA) using a GFP filter.

### Pollen fertility, viability, and germination assay

To determine pollen fertility, anthers from 30 spikelets (i.e., three spikelets each from panicles of ten individual plants) were randomly collected from RNAi, OVE, and the WT lines at the flowering stage, immediately fixed in Carnoy’s fixative solution (comprising of 99% ethanol, chloroform, and glacial acetic acid in the 6:3:1 ratio) and suspended in a 1% potassium iodide solution (I_2_-KI) at room temperature for a few minutes to stain the pollen grains. To study pollen viability, spikelets were fixed in Carnoy’s fixative solution and were stained in 1% simplified Alexander’s staining solution for 20 mins at room temperature to stain the pollen, as previously described^47^. The stained pollen grains were then observed by florescence microscopy (Life EVOS digital inverted microscopy FL, EVOS FL, Thermo Fisher Scientific Inc., USA). Pollen fertility percentage was calculated by determining the ratio of normal pollen grains to the total pollen grains per spikelet.

Pollen germination and pollen tube growth were examined using aniline blue staining as previously described^48^. Spikelets were collected within 30–60 mins following flowering and were immediately placed in a fixative solution (comprising of 99% ethanol and glacial acetic acid in the 3:1 ratio). The fixed sample was hydrated by passing through an ethanol series (70%, 50%, and 30%, and distilled water) for a duration of 10 mins for each step at room temperature. Pistils were excised, softened with 5 M NaOH at 60 °C for 1 hour and were subsequently rinsed twice for 10 mins with distilled water. Pollen tubes were stained with 0.1% (w/v) aniline blue in 100 mM K_3_PO_4_ buffer (pH 11) for 10 mins in darkness. Samples were then visualized by UV microscopy.

### Quantification of endogenous hormones and amylase

The endogenous contents of ABA, CTK, GA, IAA, and amylase were individually determined using ABA, CTK, GA, IAA, and amylase ELISA assay kits, respectively. Nine fresh rice tissues of 5-day-old RNAi and the WT seedlings were independently sampled in three replicates. These tissues were finely homogenized in a 0.01 M PBS buffer (pH 7.4). The various homogenates were centrifuged at 3,000 rpm for five minutes, and the supernatants were separately collected and preserved at −70 °C. An ELISA assay was performed according to the manufacturer’s instructions. The developed plates were analyzed to determine the various phytohormones, as well as amylase concentrations, using an automatic microplate reader (Thermo Multiskan MK3) at an absorbance of OD_450_ nm.

## Supplementary information

Supplementary Information

## Data Availability

All data generated or analyzed during this study are included in this published article (and its Supplementary Information files). The datasets generated during and/or analyzed during the current study are available from the corresponding author on reasonable request.
