# Supplementary material for: The expression pattern of OsDim1 in rice and its proposed function
Source: Sci Rep. 2019 Dec 6;9:18492. doi: 10.1038/s41598-019-54898-1 (PMC6897961; doi:10.1038/s41598-019-54898-1)
Supplement: Supplementary file 1 — Supplementary Information [file 41598_2019_54898_MOESM1_ESM.doc]

**Title: The expression pattern of *OsDim1* in rice and its proposed function**

Henry Akrofi Doku^†1,5^, Shu-Xian Gan^†1,2^, Qian Zhu^1^, Sadia Nadir^1,6^, Wei Li^1^, Meng-Ting Li^1^, Li Zhou^1^, Cheng-Yun Li^3^, Sang-Gu Kang^4^, Eui-Ho Park^4^, Li-Juan Chen^1,3*^, Dong-Sun Lee^1,3*^

^1^ Rice Research Institute, Yunnan Agricultural University, Kunming 650201, Yunnan Province, China.

^2^ Institute of Agricultural Sciences, Xishuanbanna Prefecture, Yunnan Province, China

^3^ State Key Laboratory for Conservation and Utilization of Bio-Resources in Yunnan, Yunnan Agricultural University, Kunming 650201, Yunnan, China

^4^ Department of Biotechnology, Institute of Biotechnology, College of Life and Applied Sciences, Yeungnam University, Gyeongsan, Gyeongbuk, 38541 Republic of Korea

^5^ Biotechnology Lab Complex, CSIR-Crops Research Institute, Fumesua-Kumasi, Ghana.

^6^ Department of Chemistry, University of Science and Technology Bannu, KPK, Bannu.

*Correspondence: *Li-Juan Chen 964136487@qq.com, Dong-Sun Lee dong_east@hanmail.net*

^†^ These authors contributed equally to this work

**Supplementary Table S1**. Bioinformatic analysis of the *OsDim1* promoter structure

| **Site Name** | **Oraganism** | **Sequence** | **Function** |
| --- | --- | --- | --- |
| [5UTR Py-rich stretch](http://bioinformatics.psb.ugent.be/webtools/plantcare/cgi-bin/show_site_info.htpl?QWhere=ID_of_Site like 'LE~5UTR Py-rich stretch'&StartAt=0&NbRecs=10) | Lycopersicon esculentum | TTTCTTCTCT | cis-acting element conferring high transcription levels |
| A-box | Petroselinum crispum | CCGTCC | cis-acting regulatory element |
| ABRE | Oryza sativa  Arabidopsis thaliana | GCCGCGTGGC  ACGTGGC | cis-acting element involved in the abscisic acid responsiveness |
| ARE | Zea mays | TGGTTT | cis-acting regulatory element essential for the anaerobic induction |
| ATCT-motif | Pisum sativum | AATCTAATCC | part of a conserved DNA module involved in light responsiveness |
| Box II | Petroselinum crispum | CCACGTGGC | part of a light responsive element |
| Box 4 | Petroselinum crispum | ATTAAT | part of a conserved DNA module involved in light responsiveness |
| Box-W1 | Petroselinum crispum | TTGACC | fungal elicitor responsive element |
| C-repeat/DRE | Arabidopsis thaliana | TGGCCGAC | regulatory element involved in cold- and dehydration-responsiveness |
| CAAT-box | Hordeum vulgare  Brassica rapa | CAAT  CAAAT | common cis-acting element in promoter and enhancer regions |
| CAT-box | Arabidopsis thaliana | GCCACT | cis-acting regulatory element related to meristem expression |
| CCGTCC-box | Arabidopsis thaliana | CCGTCC | cis-acting regulatory element related to meristem specific activation |
| CGTCA-motif | Hordeum vulgare | CGTCA | cis-acting regulatory element involved in the Methyl jasmonate (MeJA)-responsiveness |
| circadian | Lycopersicon esculentum | CAANNNNATC | cis-acting regulatory element involved in circadian control |
| dOCT | Arabidopsis thaliana | CaCGGATC | cis-acting regulatory element related to meristem specific activation |
| G-Box | Pisum sativum  Antirrhinum majus | CACGTG  CACGTA | cis-acting regulatory element involved in light responsiveness |
| G-box | Zea mays | CACGAC  CACGTC | cis-acting regulatory element involved in light responsiveness |
| GC-motif | Zea mays | CCCCCG | enhancer-like element involved in anoxic specific inducibility |
| GT1-motif | Avena sativa  Arabidopsis thaliana | GGTTAAT  GGTTAA | light responsive element |
| HSE | Brassica oleracea | AGAAAATTCG | cis-acting element involved in heat stress responsiveness |
| LAMP-element | Spinacia oleracea | CCAAAACCA | part of a light responsive element |
| LTR | Hordeum vulgare | CCGAAA | cis-acting element involved in low-temperature responsiveness |
| O2-site | Zea mays | GATGACATGG | cis-acting regulatory element involved in zein metabolism regulation |
| OCT | Arabidopsis thaliana | CGCGGATC | cis-acting regulatory element related to meristem specific activation |
| MBS | Arabidopsis thaliana | TAACTG | MYB binding site involved in drought-inducibility |
| MNF1 | Zea mays | GTGCCC(A/T)(A/T) | light responsive element |
| Skn-1_motif | Oryza sativa | GTCAT | cis-acting regulatory element required for endosperm expression |
| Sp1 | Zea mays | CC(G/A)CCC | light responsive element |
| TATA-box | Arabidopsis thaliana  Oryza sativa | TATA  TACAAAA | core promoter element around -30 of transcription start |
| TCCC-motif | Spinacia oleracea | TCTCCCT | part of a light responsive element |
| TGA-box | Glycine max | TGACGTGGC | part of an auxin-responsive element |
| TGA-element | Brassica oleracea | AACGAC | auxin-responsive element |
| TGACG-motif | Hordeum vulgare | TGACG | cis-acting regulatory element involved in the Methyl jasmonate (MeJA)-responsiveness |
| motif IIb | Oryza sativa | CCGCCGCGCT | abscisic acid responsive element |

**Supplementary Table S2****. List of primer sets utilized in the study**

|  | Primer name | Sequence of primer (5’ to 3’) | Restriction enzyme | Product size | Purpose |
| --- | --- | --- | --- | --- | --- |
| 1 | Os12368P-2F | GAATTCGCTTGGTTGACTTCGGCTCG | EcoRI | 3475bp | For promoter cloning |
|  | Os12368P-2R | GGATCCTGCTGCTTCTTCCTCCGCTT | BamHI |  |  |
| 2 | Os12368-1F | GGATCCCCATTCCGTTTCCCCCAAACTA | BamHI | 627bp | For full ORF cloning |
|  | Os12368-1R | ACGCGTTTAGTCAACAACACCCCACCAC | MluI |  |  |
| 3 | Os12368-1F | GGATCCCCATTCCGTTTCCCCCAAACTA | BamHI | 538bp | For protein fusion with EGFP |
|  | Os12368-3R | CCATGGTGTAACGGTATTTAGTGGAGTAGTCC | NcoI |  |  |
| 4 | Os12368Ri-1F | TGGGCCTTGAAGGACAAGCAGGAGT |  | 155bp | For RNAi1 cloning and RT PCR |
|  | Os12368Ri-1R | GGGCCACTCAACGGCAGCGT |  |  |  |
| 5 | Os12368Ri-2F | TAGCGGCAGTAGCTGAGACC |  | 331bp | For RNAi2 cloning and RT PCR |
|  | Os12368Ri-1R | GGGCCACTCAACGGCAGCGT |  |  |  |
| 6 | Actin1D-F | GTTTGAGACCTTCAACACCCCT |  | 330bp(cDNA) | For RT PCR |
|  | Actin1D-R | CTGGTCTTGGCAGTCTCCATTT |  | 579bp(gDNA) |  |

gDNA: Genomic DNA


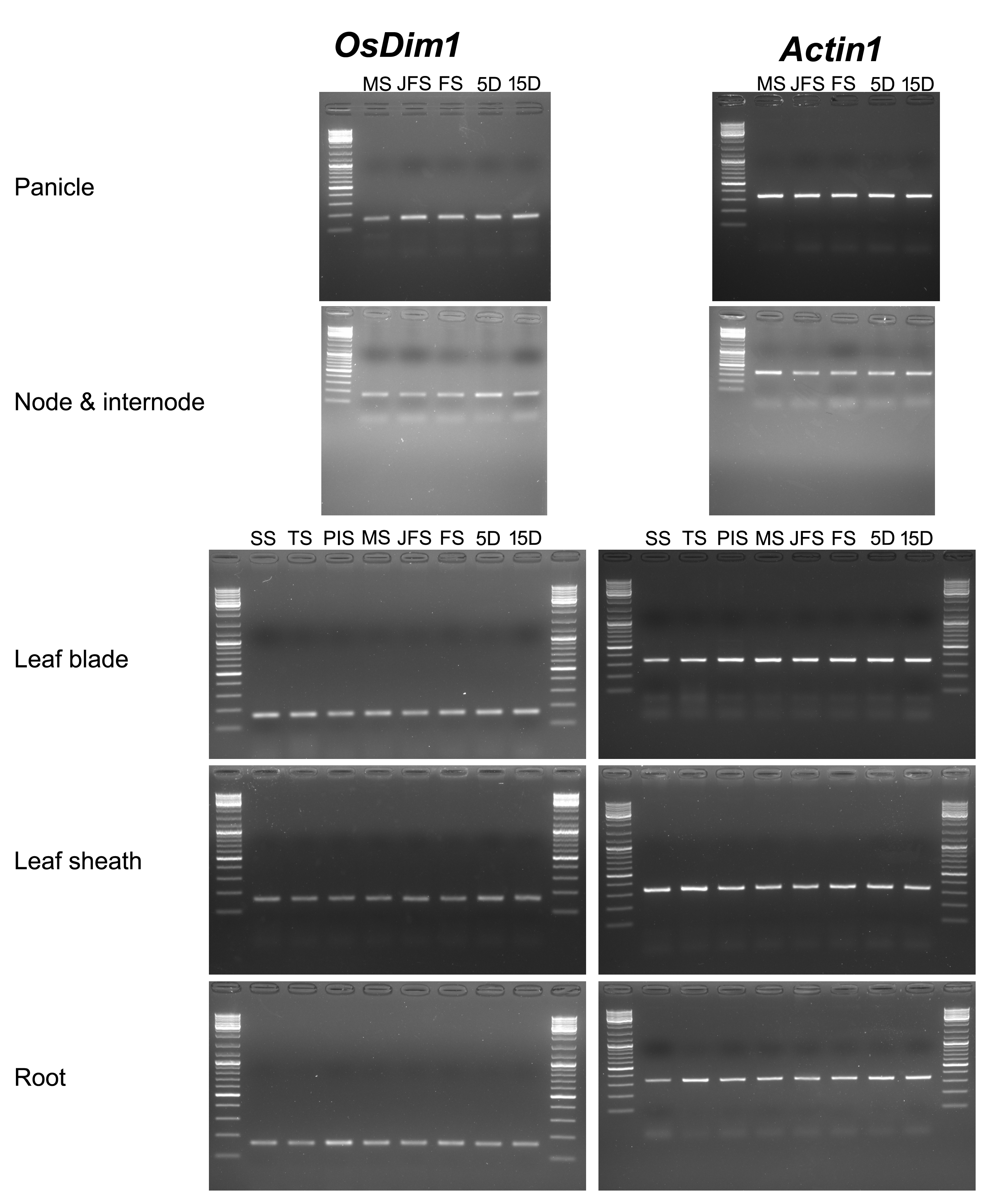


Supplementary Fig. S1. Full length gels showing RT-PCR analysis of *OsDim1* in comparison with the rice *β-actin* gene. *OsDim1* expression was detected in the panicles, nodes and internodes, leaf blade, leaf shoot and root of the rice plant at the seedling stage (SS), tiller stage (TS), panicle initiation stage (PIS), meiotic division phase of the rice panicle (flower) development (MS), just before the flowering stage (JFS), flowering stage (FS), five days after pollination (5 D), and fifteen days after pollination (15 D) of the rice plant.


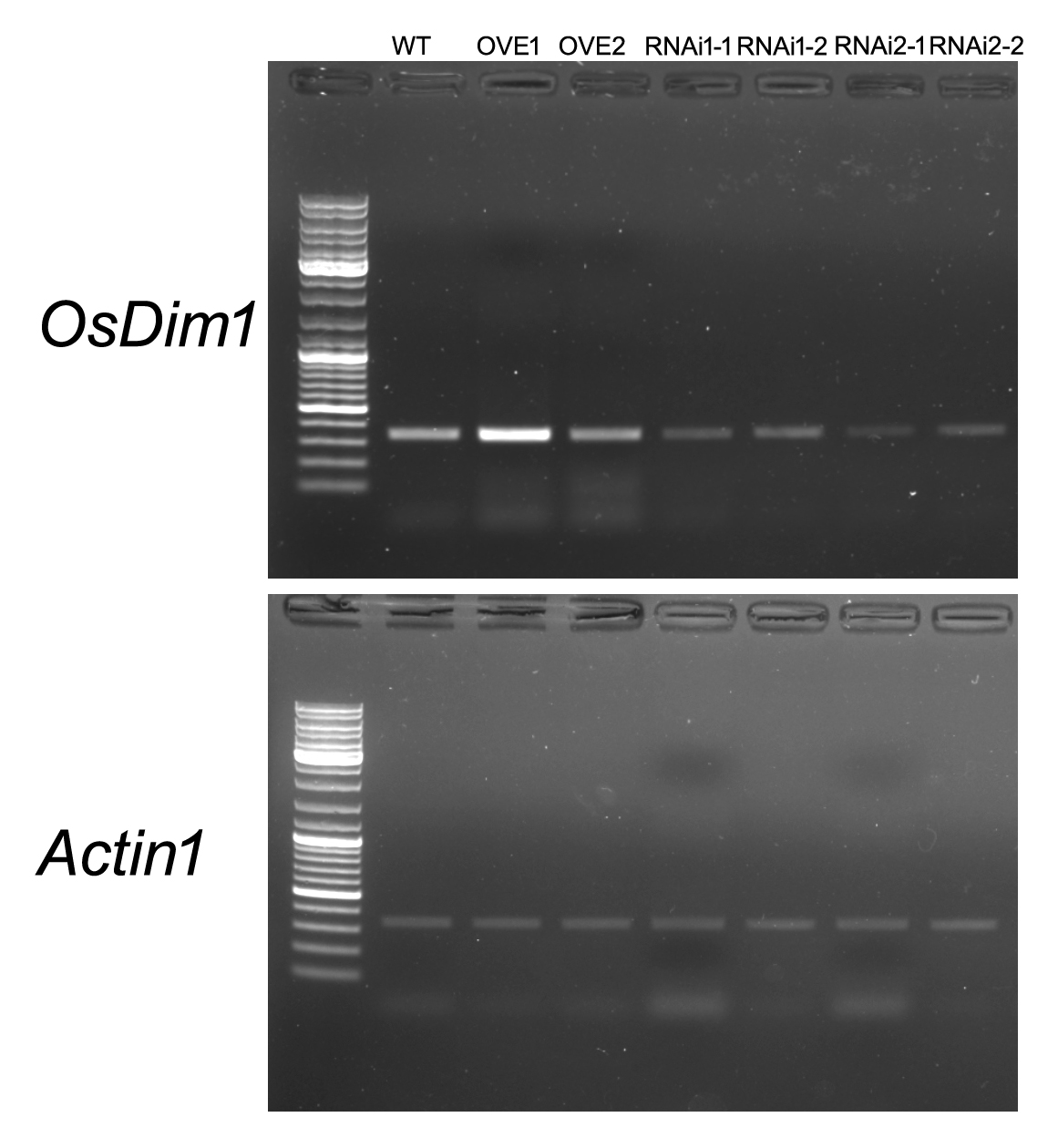


Supplementary Fig. S2. Full length gels showing RT-PCR comparison between the WT, OVE and RNAi. RT-PCR analysis showing low levels of expression of RNAi mediated knock down lines compared with the OVE and WT lines. The rice *β-actin* was used as an internal standard to normalize the expressions. Abbreviations: OVE, *OsDim1* overexpression transgenic seedlings; RNAi1, OsDim1-RNA interference (RNAi) transgenic line 1 rice seedlings; RNAi2, RNAi transgenic line 2 rice seedlings; WT, LiyuB wild type seedlings.
